# Cytomegalovirus reactivation in esophageal cancer patients receiving chemoradiotherapy: A retrospective analysis

**DOI:** 10.1002/cam4.4269

**Published:** 2021-09-13

**Authors:** Koichi Kitagawa, Hideaki Okada, Shuichiro Miyazaki, Yohei Funakoshi, Yukinari Sanada, Naoko Chayahara, Hiroshi Mayahara, Masahiko Fujii

**Affiliations:** ^1^ Kobe Minimally invasive Cancer Center Kobe Japan; ^2^ Kobe University Graduate School of Medicine Kobe Japan; ^3^ Amagasaki Chuo Hospital Amagasaki Japan; ^4^ Konan Medical Center Kobe Japan

**Keywords:** chemotherapy, clinical management, esophageal squamous cell, radiotherapy

## Abstract

**Background:**

Although rare, cytomegalovirus (CMV) reactivation can be lethal in patients with cancer. However, the criteria for the prevention of cytomegalovirus reactivation during cancer treatment are unclear. This study aimed to identify factors associated with CMV reactivation in patients with esophageal cancer who were receiving chemoradiotherapy.

**Methods:**

This retrospective study included esophageal cancer patients receiving definitive or palliative chemoradiotherapy during April 2013–March 2020. Patients with fever during chemoradiotherapy underwent a systemic work‐up to detect the primary focus of infection, and CMV antigenemia was assessed in cases of unidentifiable infection.

**Results:**

Among 132 patients (80.3% male, median age 69 years [range, 39–86 years]), 124 received 5‐fluorouracil plus cisplatin and 8 received oxaliplatin–5‐fluorouracil–levofolinate chemotherapy. Overall, 19 patients had CMV reactivation, 37 had other infections, and 76 had no identified infection (groups 1, 2, and 3, respectively). Median minimum lymphocyte counts were 81.0/µl (interquartile range: 52.0–144.0/µl), 120.0/µl (81.0–162.5/µl), and 185.5/µl (120.5–328.0/µl) in groups 1, 2, and 3, respectively, with counts being significantly lower in groups 1 and 2 than in group 3 (*p* < 0.001). In multiple logistic regression analysis, the minimum lymphocyte count was associated with CMV reactivation (odds ratio 0.983, 95% confidence interval: 0.973–0.994, *p* = 0.002).

**Conclusion:**

CMV reactivation is not rare in patients with esophageal cancer who were receiving chemoradiotherapy and is associated with the minimum lymphocyte counts. CMV reactivation should be considered during differential diagnosis for patients with a severe decline in lymphocyte counts when receiving chemoradiotherapy.

## Graphical abstract

CMV reactivation is not rare in esophageal cancer patients receiving chemoradiotherapy and is associated with minimum lymphocyte counts. CMV reactivation should be considered in differential diagnosis when a patient receiving chemoradiotherapy presents with a marked decrease in the lymphocyte count.

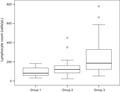

## 1 INTRODUCTION

Surgery, combined with neoadjuvant chemotherapy, is the standard therapy for operable esophageal cancer in Japan.^1^ However, older patients may have multiple comorbidities that pose difficulties in the administration of the standard therapy.^2^ Chemoradiotherapy is an alternative treatment option for patients who are ineligible to undergo surgery, although clinical management during chemoradiotherapy has proven challenging because multiple comorbidities can result in various adverse events. Most studies have focused on the efficacy of the available treatment options and major adverse events, and there is limited information on infection events among such patients.^3^

In clinical practice, cancer chemotherapy causes transient immunosuppression, with the consequent appearance of neutropenia‐related infections. Differential diagnosis of fungal infections is sometimes needed in cases where targeted antibacterial therapy is proven ineffective. Pharmacological prophylaxis of pneumocystis pneumonia and herpes zoster is recommended for patients with hematological malignancies, especially for those undergoing hematopoietic stem cell transplantation.^4^

In general, the prevalence of cytomegalovirus (CMV) infection is very high, although CMV does not affect the healthy population.^5^ Cases of CMV infection and reactivation rarely occur; therefore, they are not frequently reported in the context of cancer chemotherapy. However, CMV infection and reactivation can be fatal in immunosuppressed patients.^6^, ^7^ Therefore, prevention, surveillance, diagnosis, and treatment of CMV infection are important and generally challenging during solid organ transplantation and hematopoietic stem cell transplantation. These areas warrant more attention to reduce mortality.^8^

In our hospital, febrile events have been occasionally reported in patients with esophageal cancer who were receiving chemoradiotherapy, and their systemic work‐up identified bacterial infection and less frequently, a fungal infection. However, even after routine work‐up and treatment, some febrile events remain unresolved. Thus, in our hospital, patients with persistent fever and severe lymphopenia, such as those seen during the treatment of hematological malignancies, are examined for CMV antigenemia.

The primary aim of this study was to identify the factors associated with CMV reactivation in patients with esophageal cancer who were receiving chemoradiotherapy. The secondary objective was to analyze infectious events among patients in this study population.

## 2 MATERIALS AND METHODS

### 2.1 Patients

The clinical records of all 132 patients who received definitive chemoradiotherapy or palliative chemoradiotherapy for esophageal cancer at the Kobe Minimally invasive Cancer Center between April 2013 and March 2020 were retrospectively evaluated in this single‐center study. The study protocol complied with the principles of the Declaration of Helsinki and was approved by the institutional review board of the Kobe Minimally invasive Cancer Center (approval number: 2018‐study03‐16); the requirement for informed consent was waived because of the retrospective study design.

### 2.2 Treatments

5‐Fluorouracil plus cisplatin or oxaliplatin plus 5‐fluorouracil plus levofolinate (FOLFOX) was administered with concurrent radiotherapy. In patients treated with 5‐fluorouracil and cisplatin, two cycles were administered at a 28‐day interval. The treatment regimen involved the administration of cisplatin (70 mg/m²) on Day 1 and 5‐fluorouracil (700 mg/m²/day) as a 24‐h continuous infusion on Days 1–4, or cisplatin (75 mg/m²) on Day 1 and 5‐fluorouracil (1000 mg/m²/day) as a 24‐h continuous infusion on Days 1–4. In patients treated with FOLFOX, three cycles were administered at a 14‐day interval. The FOLFOX regimen included oxaliplatin (85 mg/m²), levofolinate (200 mg/m²), bolus 5‐fluorouracil (400 mg/m²), and 5‐fluorouracil (1600 mg/m²) infusion over 46 h. After chemoradiotherapy, two additional cycles of 5‐fluorouracil and cisplatin or three additional cycles of FOLFOX were administered to patients with stage II, III, or IV esophageal cancer.

Patients received 1.8 or 2.0 Gy/day radiation for 5 days/week, and the total radiation dose was 50.4 or 60.0 Gy, respectively.

### 2.3 Systemic work‐up for infection

In patients who developed fever during chemoradiotherapy, a systemic work‐up, including blood tests (e.g., complete blood count, bilirubin, aspartate aminotransferase, alanine aminotransferase, gamma‐glutamyl transpeptidase, lactase dehydrogenase, blood urea nitrogen, creatinine, C‐reactive protein, albumin, electrolytes, etc.), urinalysis, chest radiography, computed tomography, and blood culture as needed, was performed to detect the primary focus of infection based on the patients’ condition. In cases wherein the cause of fever could not be identified, the CMV antigen status was assessed, and CMV reactivation was diagnosed using a positive CMV antigenemia assay (C7‐HRP; SRL First and Second Hachioji Laboratory). The therapeutic effect on CMV reactivation was assessed using negative C7‐HRP test.

Adverse events were assessed according to the National Cancer Institute Common Terminology Criteria for Adverse Events (CTCAE), version 4.0. Grade 3 lymphopenia is characterized by lymphocyte counts of ≥200/mm^3^ and <500/mm^3^. Grade 4 lymphopenia is characterized by a lymphocyte count of <200/mm^3^.

### 2.4 Statistical analysis

To determine the characteristic features of CMV reactivation in the study population, the study participants were classified into the following three groups: group 1 had CMV antigenemia, regardless of the presence of other infections; group 2 had an infectious event without CMV antigenemia; and group 3 had no infection. For intergroup comparison among the three study groups, categorical data were analyzed using the Fisher's exact test, parametric continuous variables were analyzed using the unpaired *t*‐test, and nonparametric continuous variables were analyzed using the Mann–Whitney *U*‐test. The Bonferroni correction was applied for multiple comparisons. Factors with significant differences in the univariate analysis were included in the multiple logistic regression analysis to identify the factors that were associated with CMV reactivation. All statistical analyses were performed with SPSS Statistics, version 22.0 (IBM Japan, Ltd.).

## 3 RESULTS

### 3.1 Patient characteristics

Between April 2013 and March 2020, the clinical records of 132 patients with esophageal cancer, who were receiving definitive chemoradiotherapy or palliative chemoradiotherapy, were retrospectively analyzed (Table 1). Among those patients, 124 received 248 cycles of the 5‐fluorouracil plus cisplatin regimen and 8 received 24 cycles of the FOLFOX regimen. The median age at treatment initiation was 69 (range 39–86) years, and most of the patients were male (80.3%). In Japan, esophageal cancer is more prevalent in men than in women, and this was reflected in our study population as well.^3^ The most common tumor location was the middle thoracic esophagus, followed by the lower and upper thoracic esophagus. Regarding the histologic type, squamous cell carcinoma accounted for the vast majority of the cases, with only two patients presenting alternative types (undifferentiated carcinoma and adenosquamous carcinoma, respectively). Thirty‐four (25.8%) patients had metastases. The number of patients with clinical stage I, II, III, IVA, or IVB esophageal cancer was 27 (20.5%), 24 (18.2%), 22 (16.7%), 25 (18.9%), and 34 (25.8%), respectively. Eighteen (13.6%) patients received chemotherapy previously.

**TABLE 1:** Characteristics of patients in the three groups

	Group 1 (*N* = 19)	Group 2 (*N* = 37)	Group 3 (*N* = 76)	Total (*N* = 132)
Median age, years (range)	66 (47–81)	68 (54–84)	69 (39–86)	69 (39–86)
Sex				
Male	13 (68.4)	31 (83.8)	62 (81.6)	106 (80.3)
Female	6 (31.6)	6 (16.2)	14 (18.4)	26 (19.7)
PS (ECOG)				
0	1 (5.3)	11 (29.7)	29 (38.2)	41 (31.1)
1	17 (89.5)	24 (64.9)	40 (52.6)	81 (61.4)
2	1 (5.3)	1 (2.7)	7 (9.2)	9 (6.8)
3	0 (0)	1 (2.7)	0 (0)	1 (0.8)
Clinical T stage				
1	2 (10.5)	6 (16.2)	20 (26.3)	28 (21.2)
2	2 (10.5)	7 (18.9)	13 (17.1)	22 (16.7)
3	6 (31.6)	16 (43.2)	29 (38.2)	51 (38.6)
4	9 (47.4)	8 (21.6)	14 (18.4)	31 (23.5)
Clinical N stage				
N0	4 (21.1)	10 (27.0)	28 (36.8)	42 (31.8)
N1	6 (31.6)	12 (32.4)	24 (31.6)	42 (31.8)
N2	4 (21.1)	10 (27.0)	16 (21.1)	30 (22.7)
N3	5 (26.3)	5 (13.5)	8 (10.5)	18 (13.6)
Clinical M stage				
M0	12 (63.2)	30 (81.1)	56 (73.7)	98 (74.2)
M1	7 (36.8)	7 (18.9)	20 (26.3)	34 (25.8)
Clinical stage				
I	2 (10.5)	6 (16.2)	19 (25.0)	27 (20.5)
II	2 (10.5)	6 (16.2)	16 (21.1)	24 (18.2)
III	1 (5.3)	11 (29.7)	10 (13.2)	22 (16.7)
IVA	7 (36.8)	7 (18.9)	11 (14.5)	25 (18.9)
IVB	7 (36.8)	7 (18.9)	20 (26.3)	34 (25.8)
Location of the primary tumor				
Ce	2 (10.5)	1 (2.7)	10 (13.2)	14 (10.6)
Ut	4 (21.1)	7 (18.9)	14 (18.4)	25 (18.9)
Mt	6 (31.6)	15 (40.5)	36 (47.4)	57 (43.2)
Lt	1 (5.3)	13 (35.1)	14 (18.4)	28 (21.2)
EGJ	0	0	1 (1.3)	1 (0.8)
Multiple areas	6 (31.6)	1 (2.7)	0	7 (5.3)
Histological type				
Squamous cell carcinoma	19 (100)	37 (100)	74 (97.4)	130 (98.5)
Others	0	0	2 (2.6)	2 (1.5)
Prior chemotherapy				
Yes	2 (10.5)	6 (16.2)	8 (10.5)	18 (13.6)
No	17 (89.5)	31 (83.8)	68 (89.5)	114 (86.4)
Comorbidity				
Diabetes mellitus	3 (15.8)	3 (8.1)	12 (15.8)	18 (13.6)

Abbreviations: Ce, cervical esophagus; ECOG PS, Eastern Cooperative Oncology Group performance status; EGJ, esophago‐gastric junction; Lt, lower thoracic esophagus; Mt, middle thoracic esophagus; Ut, upper thoracic esophagus.

### 3.2 Infection‐related events

Patients were classified into three study groups according to infection‐related events, as described earlier (Table 1). CMV antigenemia occurred mainly after the second cycle of chemotherapy (*n* = 17), with the most common manifestation of CMV infection being fever (Table 2). In patients without CMV reactivation (group 2), most infection‐related events occurred after the second chemotherapy cycle (Table 3). The main cause of infection was bacterial esophagitis, with other infections (such as pneumonia, colitis, febrile neutropenia, or fungal infection) being less frequent.

**TABLE 2:** Infection in group 1

Characteristics	Cycle 1 (*N* = 19)	Cycle 2 (*N* = 19)	Cycle 3 (*N* = 2)
CMV antigenemia	1	17	1
Chemotherapy regimen			
Fluorouracil and Cisplatin	17	17	0
FOLFOX	2	2	2
Symptoms of CMV reactivation			
None (fever only)	1	11	0
Pneumonia	0	3	0
Hepatitis	0	3	1
Colitis	0	1	0
Infection status			
No infection	10	0	1
Bacterial esophagitis	4	4	0
Febrile neutropenia	2	1	0
Fungal infection	1	2	0
Esophago‐tracheal fistula	1	1	0

Abbreviations: CMV, cytomegalovirus; FOLFOX, oxaliplatin and infused fluorouracil plus levofolinate.

**TABLE 3:** Infection in group 2

Characteristics	Cycle 1 (*N* = 37)	Cycle 2 (*N* = 37)	Cycle 3 (*N* = 3)
Chemotherapy regimen			
Fluorouracil and CDDP	34	34	0
FOLFOX	3	3	3
Infection			
None	21 (56.8)	9 (24.3)	0
Bacterial esophagitis	9 (24.3)	22 (59.5)	3 (100)
Pneumonia	1 (2.7)	2 (5.4)	0
Hepatitis	0	0	0
Colitis	0	1 (2.7)	0
Febrile neutropenia	1 (2.7)	1 (2.7)	0
Fungal infection	0	1 (2.7)	0
Others	5 (13.5)	1 (2.7)	0

Abbreviations: CDDP, cisplatin; FOLFOX, oxaliplatin and infused fluorouracil plus levofolinate.

### 3.3 Association of patient characteristics with infection

A comparison of patient characteristics, such as age, sex, performance status, body mass index (BMI), clinical TNM staging, clinical stage, tumor location, presence of diabetes mellitus, and pretreatment chemotherapy among groups 1, 2, and 3 is shown in Table 4; no significant intergroup differences, except for tumor location, were noted. The incidence of lower thoracic tumors was lower in group 1 than in groups 2 and 3. However, the incidence of tumor location in multiple areas was higher in group 1 than in groups 2 and 3 (*p* = 0.019 for tumor location in the three study groups).

**TABLE 4:** Association of patient characteristics and variables with infection

Characteristics	Group 1 (*N* = 19)	Group 2 (*N* = 37)	Group 3 (*N* = 76)	*p*‐value for all	*p*‐value of Group 1 versus Group 2	*p*‐value of Group 1 versus Group 3	*p*‐value of Group 2 versus Group 3
Age	66.7 ± 7.8	68.2 ± 6.9	68.8 ± 9.2	0.637^b^	>0.999^e^	>0.999^e^	>0.999^e^
Sex				0.373^a^	0.905^d^	0.660^d^	>0.999^d^
Male	13, 68.4	31, 83.8	62, 81.6				
Female	6, 31.6	6, 16.2	14, 18.4				
PS				0.095^c^	0.215^f^	0.101^f^	>0.999^f^
0	1, 5.3	11, 29.7	29, 38.2				
1	17, 89.5	24, 64.9	40, 52.6				
2	1, 5.3	1, 2.7	7, 9.2				
3	0, 0.0	1, 2.7	0, 0.0				
BMI	20.5 ± 2.5	20.9 ± 3.0	20.6 ± 3.1	0.861^b^	>0.999^e^	>0.999^e^	>0.999^e^
Clinical T stage				0.037^c^	0.237^f^	0.038^f^	0.995^f^
1	2, 10.5	6, 16.2	20, 26.3				
2	2, 10.5	7, 18.9	13, 17.1				
3	6, 31.6	16, 43.2	29, 38.2				
4	9, 47.4	8, 21.6	14, 18.4				
Clinical *N* stage				0.184^c^	>0.999^f^	0.274^f^	0.798^f^
0	4, 21.1	10, 27.0	28, 36.8				
1	6, 31.6	12, 32.4	24, 31.6				
2	4, 21.1	10, 27.0	16, 21.1				
3	5, 26.3	5, 13.5	8, 10.5				
Clinical M stage				0.358^a^	0.585^d^	>0.999^d^	>0.999^d^
0	12, 63.2	30, 81.1	56, 73.7				
1	7, 36.8	7, 18.9	20, 26.3				
Clinical stage				0.105^c^	0.154^f^	0.147^f^	>0.999^f^
I	2, 10.5	6, 16.2	19, 25.0				
II	2, 10.5	6, 16.2	16, 21.1				
III	1, 5.3	11, 29.7	10, 13.2				
IVA	7, 36.8	7, 18.9	11, 14.5				
IVB	7, 36.8	7, 18.9	20, 26.3				
Tumor location				0.019^a^	0.041^d^	0.320^d^	0.287^d^
Ce	2, 10.5	1, 2.7	11, 14.5				
Lt	1, 5.3	14, 37.8	13, 17.1				
Mt	8, 42.1	14, 37.8	35, 46.1				
EGJ	0, 0.0	0, 0.0	1, 1.3				
Ut	4, 21.1	7, 18.9	14, 18.4				
Multiple areas	4, 21.1	1, 2.7	2, 2.6				
Diabetes mellitus				0.491^a^	>0.999^d^	>0.999^d^	>0.999^d^
No	16, 84.2	34, 91.9	64, 84.2				
Yes	3, 15.8	3, 8.1	12, 15.8				
Pretreatment chemotherapy				0.719^a^	>0.999^d^	>0.999^d^	>0.999^d^
Yes	3, 15.8	6, 16.2	9, 11.8				
No	16, 84.2	31, 83.8	67, 88.2				
Pretreatment Hb	11.8 ± 1.5	12.4 ± 1.6	12.6 ± 1.8	0.159^b^	0.485^e^	0.192^e^	>0.999^e^
Pretreatment Alb	3.6 ± 0.5	3.7 ± 0.5	3.9 ± 0.6	0.126^b^	>0.999^e^	0.190^e^	0.713^e^
Pretreatment Lym	1346.4 ± 333.8	1425.8 ± 435.4	1448.6 ± 495.7	0.687^b^	>0.999^e^	>0.999^e^	>0.999^e^
Pretreatment WBC	8268.4 ± 5292.2	6967.6 ± 4772.7	6601.3 ± 2781.0	0.243^b^	>0.999^e^	0.180^e^	>0.999^e^
PTV (cc)	559.0 [464.9, 747.9]	567.8 [457.5, 677.9]	459.5 [320.7, 599.3]	0.005^c^	>0.999^f^	0.023^f^	0.037^f^
Minimum lymphocyte count	81.0 [52.0, 144.0]	120.0 [81.0, 162.5]	185.5 [120.5, 328.0]	**<**0.001^c^	0.216^f^	<0.001^f^	<0.001^f^
Minimum neutrophil count	900.0 [600.0, 1200.0]	1000.0 [700.0, 1350.0]	1200.0 [925.0, 1600.0]	0.005^c^	0.810^f^	0.018^f^	0.060^f^
RDI of CDDP or L‐OHP (%)	96.6 [83.7, 99.8]	98.7 [86.2, 100.3]	98.0 [88.7, 100.0]	0.723^c^	>0.999^f^	>0.999^f^	>0.999^f^
RDI of 5‐FU (%)	99.3 [90.4, 100.4]	99.9 [98.7, 101.4]	99.5 [96.0, 100.8]	0.323^c^	0.556^f^	>0.999^f^	0.727^f^
Irradiated dose (Gy)	60.0 [60.0, 60.0]	60.0 [54.0, 60.0]	60.0 [60.0, 60.0]	0.545^c^	>0.999^f^	>0.999^f^	0.914^f^

Note

Categorical data, including sex, PS, clinical TNM staging, clinical stage, tumor location, diabetes mellitus, and pretreatment chemotherapy, were analyzed using Fisher's exact test. Parametric continuous variables, including age, BMI, Hb, Alb, Lym, and WBC, were analyzed using unpaired *t*‐test. Nonparametric continuous variables, including the minimum lymphocyte count, minimum neutrophil count, RDI of CDDP or L‐OHP, RDI of 5‐FU, and the irradiated dose, were analyzed using the Mann–Whitney *U*‐test. The Bonferroni correction was applied for multiple comparisons.

Data are expressed as *n*, % for categorical data; mean ± SD for parametric data; and median [IQR] for nonparametric data. *P*‐values were obtained using the: ^a^Fisher's exact test (for all); ^b^one‐way analysis of variance; ^c^Kruskal–Wallis test; ^d^Fisher's exact test (Bonferroni correction); ^e^unpaired *t*‐test (Bonferroni correction); ^f^Mann–Whitney *U*‐test (Bonferroni correction).

Abbreviations: 5‐FU, 5‐fluorouracil; Alb, pretreatment albumin; BMI, body mass index; CDDP, cisplatin; Ce, cervical esophagus; EGJ, esophago‐gastric junction; Hb, hemoglobin; IQR, interquartile range; L‐OHP, oxaliplatin; Lt, lower thoracic esophagus; Lym, pretreatment lymphocyte count; Mt, middle thoracic esophagus; PS, performance status; PTV, planning target volume; RDI, relative dose intensity; Ut, upper thoracic esophagus; WBC, pretreatment white blood cell count.

### 3.4 Association of pretreatment variables with infection

**FIGURE 1:**
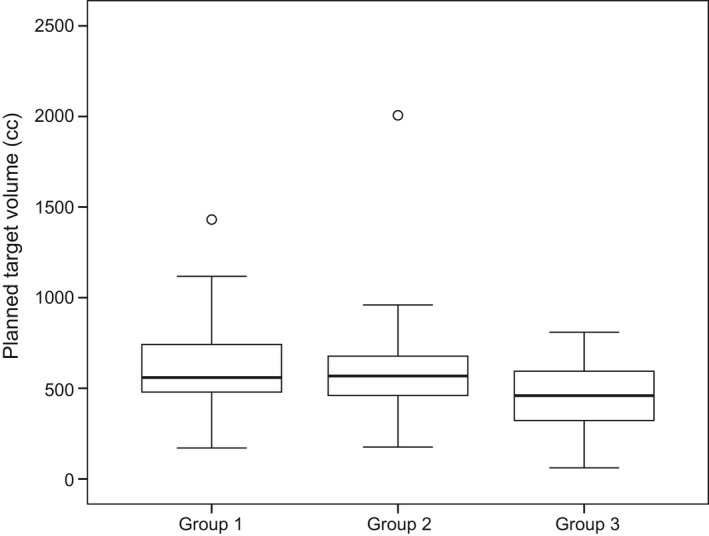
Planning target volume (PTV; cc) in the different study groups. PTV in groups 1 and 2 was significantly larger than that in group 3 (*p* = 0.023 for group 1 vs. group 3 and *p* = 0.037 for group 2 vs. group 3, using the Mann–Whitney *U*‐test with Bonferroni correction). However, the difference in PTV between groups 1 and 2 was not significant (*p* > 0.999, using the Mann–Whitney *U*‐test with Bonferroni correction). For each boxplot, the box limits represent the 25th and 75th percentiles, the line within each box represents the median, and the whisker ends indicate the 10th and 90th percentiles

Pretreatment hemoglobin levels, albumin levels, lymphocyte counts, white blood cell (WBC) counts, and planning target volume (PTV) were compared among groups 1, 2, and 3 (Table 4). Only PTV showed a significant difference among the three groups: PTV in groups 1 and 2 was significantly larger than that in group 3; however, the difference between groups 1 and 2 in this regard was not significant (Figure 1).

### 3.5 Association between variables during treatment period and infection

**FIGURE 2:**
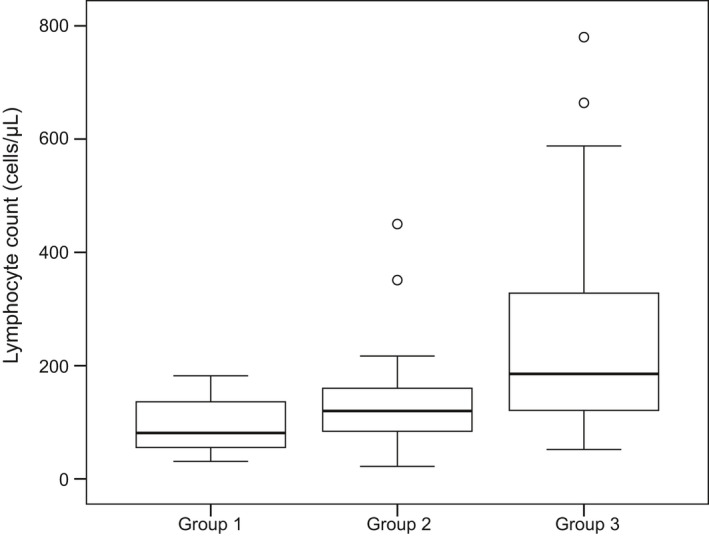
Minimum lymphocyte count in the study groups. The minimum lymphocyte counts in groups 1 and 2 were significantly lower than those in group 3 (*p* < 0.001 for both comparisons, using the Mann–Whitney *U*‐test with Bonferroni correction). However, the difference between groups 1 and 2 was not significant (*p* = 0.216, using the Mann–Whitney *U*‐test with Bonferroni correction). For each box plot, the box limits represent the 25th and 75th percentiles, the line within each box represents the median, and the whisker ends indicate the 10th and 90th percentiles

Grade 3 and 4 lymphopenia were observed in 19 (100%), 31 (83.8%), and 40 (52.6%) patients in groups 1, 2, and 3, respectively, implying that all patients experienced Grade 3 lymphopenia that subsequently progressed to Grade 4 lymphopenia. The minimum lymphocyte count, minimum neutrophil count, relative dose intensity (RDI) of cisplatin or oxaliplatin, RDI of 5‐fluorouracil, and irradiated dose were compared among the three study groups (Table 4). The minimum lymphocyte count in groups 1 and 2 was significantly lower than that in group 3, although the difference between groups 1 and 2 was not significant (Figure 2). The minimum neutrophil count in group 1 was significantly lower than that in group 3; however, the difference between groups 1 and 2 was not significant. There was no significant intergroup difference in the RDI of chemotherapy and irradiated dose among the three study groups.

### 3.6 Multivariate analysis to detect factors associated with CMV reactivation

Multiple logistic regression analysis revealed that only the minimum lymphocyte count was associated with CMV reactivation (odds ratio 0.983, 95% confidence interval: 0.973–0.994, *p* = 0.002).

This odds ratio represents the risk of developing CMV reactivation when the lymphocyte count increases by one. Therefore, the odds ratio when the lymphocyte count decreases by one is the reciprocal, and the odds ratio when the lymphocyte count decreases by X is the reciprocal to the Xth power.

## 4 DISCUSSION

To the best of our knowledge, this is the first study to identify the factors associated with CMV reactivation in patients undergoing cancer treatment and show that chemoradiotherapy‐induced lymphopenia was associated with CMV reactivation. To date, the risks of CMV infection have been discussed with respect to HIV infection, organ transplantation, and hematopoietic stem cell transplantation, and no study has clarified the relationship between lymphocyte count and CMV reactivation in cancer chemotherapy.

Based on the degree of neutropenia after chemotherapy, an associated increase in the risk of bacterial infection has been reported.^9^ Therefore, clinical guidelines have described the treatment strategy for fever during chemotherapy.^10^ Febrile neutropenia, defined as ANC <1000/mm^3^ with a single temperature of >38.3℃ or a sustained temperature of >=38℃ for more than 1 h, has an established standard treatment.^11^, ^12^ However, the management of infection depending on the degree of lymphopenia has not been fully established. In chemotherapy for solid cancers, CMV infection and reactivation occurs rarely, and CMV surveillance is not routinely performed in clinical practice.

In our hospital, we observed that patients with persistent fever and no obvious focus of infection usually had concurrent lymphopenia, similar to what occurs in patients undergoing chemotherapy for hematological malignancies. Therefore, we decided to start testing for CMV in this population. Initially, it was hypothesized that chemotherapy had a large effect on lymphopenia. However, contrary to our expectations, the RDI of chemotherapy in the three study groups showed no apparent difference. Next, we explored possible risk factors for lymphopenia in the pretreatment patient characteristics, including age, sex, performance status, BMI, clinical TNM staging, clinical stage, tumor location, diabetes mellitus, hemoglobin, albumin, lymphocytes, and WBC. However, none of these factors were apparently related to lymphopenia.

Some studies have revealed an inverse correlation between PTV and the lymphocyte count in radiation therapy for esophageal cancer.^13^, ^14^ However, these studies did not report whether PTV‐induced lymphopenia resulted in infection. In this study, we confirmed an inverse correlation between PTV and the lymphocyte count. As the RDI in the three groups did not show a significant difference, the differences in the decrease in lymphocyte counts could be attributed to the degree of PTV. Therefore, additional lymphopenia caused by radiation in addition to baseline lymphopenia caused by chemotherapy increased the risk of infection, including that of CMV reactivation.

This study had some limitations. First, patients in this retrospective study did not undergo pretreatment testing for CMV. In general, CMV asymptomatically infects the host during childhood and establishes life‐long latency.^8^ Moreover, CMV seroprevalence is very high in Japan.^5^ In addition to these factors, all patients were hospitalized to receive chemoradiotherapy, and there was no possibility that they could have contracted a primary CMV infection because they did not have the opportunity to come into contact with body fluids, including saliva, urine, and genital secretions contaminated with the virus. Based on the abovementioned reasons, CMV antigenemia was interpreted as CMV reactivation.

Second, pneumonia, hepatitis, and colitis were diagnosed clinically as CMV infection based on symptoms, laboratory test results, and imaging findings. To obtain a precise and definitive diagnosis of CMV infection, a biopsy is necessary for histological confirmation, including hematoxylin and eosin, immunoperoxidase, and/or immunofluorescence staining, or in‐situ hybridization using a DNA probe.^15^

However, in patients who were exhausted owing to chemoradiotherapy, a biopsy was deemed too difficult to perform. Even if examinations, such as gastroscopy, bronchoscopy, and biopsy, are feasible, there is a concern that the associated delay may prove life‐threatening owing to the late initiation of treatment.^6^

CMV esophagitis has been reported as a cause of fever during chemoradiotherapy for esophageal cancer.^16^, ^17^ In these reports, CMV infection was confirmed using tissue biopsy. However, this method is not highly sensitive because the main location of latent CMV infection is myeloid progenitor cells, and the true nature of CMV reactivation is viral replication in granulocytes and macrophage cells under immunosuppression.^18^, ^19^, ^20^

Given that DNA transcription is completely regulated during latent CMV infection,^21^ the presence of antigenemia is sufficient to diagnose viral reactivation. In the early stages of CMV reactivation, fever can be caused by elevated TNF‐α and IFN‐γ levels, and CMV antigenemia may be the sole specific finding, with the virus not yet being detectable in the suspected infected tissue.^7^ Even when CMV is not detectable using biopsy, the presence of symptoms and a positive CMV antigenemia should prompt treatment initiation to prevent serious organ damage. In fact, treatment with ganciclovir resulted in negative CMV antigenemia in all patients in this retrospective study and prevented death due to CMV infection.

In conclusion, this retrospective study demonstrated that lymphopenia caused by chemoradiation was associated with CMV reactivation, and that PTV had a greater effect on lymphopenia than chemotherapy. In patients undergoing treatments that induce severe lymphopenia, CMV reactivation should be considered during the differential diagnosis of persistent fever. This may allow early diagnosis and treatment, as well as prevent serious complications and death.

## CONFLICT OF INTEREST

The authors declare that they have no conflict of interest.

## ETHICAL APPROVAL

All patients who received definitive chemoradiotherapy or palliative chemoradiotherapy from April 2013 to March 2020 were included and treated at the Kobe Minimally invasive Cancer Center. The study protocol complied with the principles of the Declaration of Helsinki and was approved (approval number: 2018‐study03‐16) by the institutional review board of Kobe Minimally invasive Cancer Center, which waived the need for written informed consent because of the retrospective study design.

## Data Availability

Data available upon request from the authors.
